# The effect of surgically induced ischaemia on gene expression in a colorectal cancer xenograft model

**DOI:** 10.1038/sj.bjc.6602905

**Published:** 2005-12-13

**Authors:** G Atkin, F M Daley, S Bourne, R Glynne-Jones, J Northover, G D Wilson

**Affiliations:** 1 Gray Cancer Institute, Mount Vernon Hospital, Northwood, Middlesex HA6 2RN, UK; 2 Department of Radiotherapy, Mount Vernon Hospital, Northwood, Middlesex HA6 2RN, UK; 3 Colorectal Cancer Unit, St Mark's Hospital, Harrow HA1 3UJ, UK; 4 Karmanos Cancer Institute, Wayne State University, Detroit, MI 48201-2013, USA

**Keywords:** gene expression, hypoxia, thymidylate synthase

## Abstract

Delays in tissue fixation following tumour vascular clamping and extirpation may adversely affect subsequent protein and mRNA analysis. This study investigated the effect of surgically induced ischaemia in a xenograft model of a colorectal cancer on the expression of a range of prognostic, predictive, and hypoxic markers, with a particular emphasis on thymidylate synthase. Vascular occlusion of human tumour xenografts by D-shaped metal clamps permitted defined periods of tumour ischaemia. Alterations in protein expression were measured by immunohistochemistry and spectral imaging, and changes in mRNA were measured by reverse transcriptase–polymerase chain reaction. Thymidylate synthase expression decreased following vascular occlusion, and this correlated with cyclin A expression. A similar reduction in dihydropyrimidine dehydrogenase was also seen. There were significant changes in the expression of several hypoxic markers, with carbonic anhydrase-9 showing the greatest response. Gene transcriptional levels were also noted to change following tumour clamping. In this xenograft model, surgically induced tumour ischaemia considerably altered the gene expression profiles of several prognostic and hypoxic markers, suggesting that the degree of tumour ischaemia should be minimised prior to tissue fixation.

During the surgical resection of a solid tumour, the principal arterial supply is clamped in order to obtain vascular control and thereby facilitate the subsequent dissection and tumour removal. This vascular clamping and extirpation renders the tumour ischaemic, during which time tissue proteins undergo proteolytic degradation until fixed in formalin or liquid nitrogen. Delays in fixation reduce the efficacy of subsequent protein and mRNA analysis (Werner *et al*, 2000; Almeida *et al*, 2004), and it has been suggested that tissue fixation should occur within 30 min of surgical extirpation (Werner *et al*, 2000). However, there are no formal clinical guidelines regarding the rapidity of fixation, hence the ischaemic insult may be prolonged unduly.

Hypoxia and acidosis, both of which occur during tumour vascular clamping (Parkins *et al*, 1997), are potent mediators of gene expression (Helfman and Falanga, 1993). Warm ischaemia following extirpation alters the gene expression profile of a range of tissue types (Miyatake *et al*, 2004), and changes in gene expression have been seen in colorectal mucosa commencing 20 min after extirpation (Huang *et al*, 2001). It was hypothesized, therefore, that in addition to the effect of degradation following excision, surgically induced ischaemia occurring during colorectal cancer (CRC) surgery may have a significant impact on gene expression levels, thereby altering the expression profiles of postoperative tumour samples compared with *in situ* levels.

A range of biomarkers have been investigated as estimates of prognosis and response to chemotherapy in a number of tumour types. Thymidylate synthase (TS) has been the most extensively studied prognostic and predictive marker in CRC (Adlard *et al*, 2002). It catalyses the reaction that provides the thymidine nucleotide needed for DNA synthesis, and is the main site of action of 5-fluorouracil (5-FU) (van der Wilt and Peters, 1994). High tumour TS levels have been consistently associated with a poor prognosis (Edler *et al*, 2000; Takenoue *et al*, 2000), whereas low TS levels favour a better outcome to 5-FU-based chemotherapy (Okonkwo *et al*, 2001; Aschele *et al*, 2002). However, studies investigating TS have shown methodological discrepancies, with levels being measured in preoperative biopsies and postoperative archival tumour sections (Edler *et al*, 2000; Okonkwo *et al*, 2001). If surgery alters biomarker levels, this would have major implications on the timing of marker measurement in relation to the surgical procedure. Hence, the aim of this study was to investigate the effect of surgically induced ischaemia on marker levels in a xenograft model of CRC, looking in particular at TS.

## MATERIALS AND METHODS

**Figure 1:**
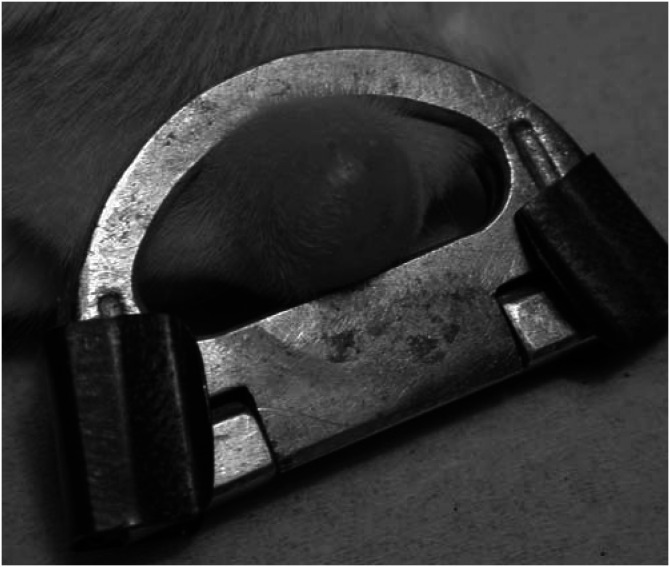
D-clamps used to occlude tumour vascular inflow.

Subcutaneous tumours were generated by injecting human HT-29 cells in the dorsal flank of 55 female severe combined immunodeficiency (SCID) mice, and allowed to grow to a diameter of 0.5 cm (approximately 3 weeks). D-shaped metal clamps, which have been shown to occlude 99.9% of the vascular inflow (Denekamp *et al*, 1983), were used to induce vascular occlusion in the subcutaneous tumours for time periods of 0, 1, 2, 4, 8, 12, 16, 20, and 24 h (Figure 1). Five mice were randomly allocated to each time period. Time point zero (no clamping) was used as an internal control. All experimental work was carried out in accordance with Home Office guidelines.

### Immunohistochemistry

Table 1 shows the proteins and the conditions used for immunohistochemistry. Sections (4 *μ*m) were dewaxed in xylene for 5 min and rehydrated through graded alcohol (100, 90, and 70%) to water. Heat-mediated antigen retrieval was performed using 250 ml 10 mM citric acid, pH 6, for all markers, apart from vascular endothelial growth factor (VEGF) (0.1 M Tris-HCL pH10) and carbonic anhydrase-9 (CA-9) (no pretreatment), by boiling the sections in an 800 W microwave oven (Panasonic NN-6453BBPQ, 2450 MHz).

For all markers apart from CA-9 and hypoxia-inducible factor-1 (HIF-1)*α*, sections were transferred to the DAKO Autostaining machine (DAKO, UK) containing peroxidase block (DAKO, S2023), the detection reagents (ChemMate HRP, DAKO K5001), and anti-human primary antibody diluted in antibody diluent. The Autostainer programme included 5 min in peroxidase block, 1 h incubation in primary antibody, 30 min incubation in ChemMate secondary and tertiary reagents, and 5 min in diaminobenzidine (DAB) substrate. An additional step was then performed to mask the xenograft epitopes and reduce background staining. This was achieved with Ultravision Rodent Block (Lab Vision Corporation, Fremont, USA).

Sections were stained for HIF-1*α* using the DakoCytomation CSA II signal amplification system (DAKO Corporation, Carpinteria, USA). In summary, the sections were first incubated with 3% hydrogen peroxide for 5 min to quench endogenous peroxidase activity, following which incubation with a protein block for 5 min was performed to inhibit nonspecific binding. Diluted primary antibody was added and sections were incubated for 15 min. Sequential 15 min incubations were performed with anti-mouse Ig-HRP, fluorescyl-tyramide hydrogen peroxide, and anti-fluorescein-HRP. Finally, the slides were incubated for 5 min with DAB/hydrogen peroxide. For CA-9, endogenous peroxidase activity was blocked using DAKO peroxidase block (Envision kit) for 5 min. Then, a DAKO Protein Block (X0909) was added for a further 5 min, following which incubation with the CA-9 primary antibody diluted 1/50 in tris-buffered saline (TBS) for 30 mins was performed . A further incubation was then performed with DAKO Envision HRP Mouse polymer (K4006) for 30 min, followed by 5 min with DAB solution.

Each staining run incorporated a control slide that had previously demonstrated positive for the antibody of interest. A negative control was also incorporated and involved the substitution of the anti-human primary antibody for an isotypic control antibody at the same protein concentration.

### Quantification of marker protein expression by spectral imaging

Immunohistochemical staining of marker protein expression was quantified using a spectral imager developed and constructed in our Institute, as reported previously (Barber *et al*, 2003). This allowed accurate immunostain quantification, with stain intensity being expressed as optical density (OD) normalized to reference spectra. Most markers demonstrated greater expression in tumour cells compared with stroma and by applying arbitrary thresholds to the OD data, nonspecific background staining could be minimized on the basis of its lower OD, thus providing exclusive quantification of marker protein expression within tumour tissue. For markers exhibiting similar tumour and stromal staining, a threshold was chosen that included the staining of both tissue compartments; however, the same threshold was applied to all images for each marker, thereby minimizing the error of background staining. In addition, the number of pixels with stain intensity above the threshold was determined and represented the area of the captured image demonstrating marker expression. Each captured image had a high tumour/stroma ratio, hence the total tumour area was uniform between captured images and allowed comparison of positively stained area.

Three images were captured using spectral imaging for each tumour at each time point. Mean stain intensity and area were determined for each captured image, giving 15 values for each time point. The data were normalized by taking the ratio of the mean at the observation time relative to that at time zero. For cyclin A, images were captured in a similar way; however, a labelling index (defined as the ratio of positive to negative nuclei) was manually calculated for each section and a mean at each duration of ischaemia was expressed relative to time point zero. Hypoxia-inducible factor-1 (HIF-1)*α* expression, which showed mixed nuclear and cytoplasmic staining, was determined by spectral imaging and labelling index calculation.

In order to assess the overall trend in expression for each marker, the area under the curve (AUC) was calculated for the intensity and area expression profiles of each marker. The change in expression relative to the basal level was determined by subtracting 24 (the AUC if there was no change in expression) from the marker AUC calculation.

### Reverse transcriptase–polymerase chain reaction (RT–PCR)

Tumour samples were removed from frozen storage (−70°C) and transferred on ice. Total RNA isolation was performed using the RNeasy® Mini Kit (Qiagen Ltd, UK). Thymidylate synthase and HIF-1*α* RT–PCR was performed using the Platinum® Quantitative RT–PCR Thermoscript™ One-Step System (Invitrogen™ Ltd, Paisley, UK). *β*-Actin (*β*A) was used as an internal control. The RT–PCR conditions were as follows: 75 ng of tumour RNA was used for each reaction in a final volume of 50 *μ*l, containing 150 pmol of each TS primer and 75 pmol for each set of HIF-1*α* and *β*A primers, 25 *μ*l 2 × Thermoscript™ Reaction buffer, and 1 *μ*l of Thermoscript™ Plus/Platinum® *Taq* Mix. The sequences of the oligonucleotide primers have been reported previously for TS (Leichman *et al*, 1997), HIF-1*α* (Kuwai *et al*, 2003), and *β*A (Leichman *et al*, 1997). The following amplification protocol was used: cDNA synthesis at 60°C for 30 min, denaturation at 95°C for 5 min, followed by 28 cycles (for TS and BA) or 25 cycles (for HIF-1*α*) of 95°C for 20 s, 56°C for 30 s, and 70°C for 30 s. Finally, the PCR products were elongated at 70°C for 2 min.

The reaction mixtures were subsequently applied to a 1.8% agarose gel and the amplified products were stained with ethidium bromide. Following electrophoresis, band intensity was measured by ultraviolet (UV) densitometry using a custom-built image capture system consisting of an eight-bit charge-coupled device (CCD) camera (COHU, Brian Reece Scientific, UK), image capture board (Data Translations, Basingstoke, UK) DT55–50 Hz, eight-bit 786 × 512 pixels image capture), and an in-house developed analysis package (based on Visilog software, Datacell, UK). Thymidylate synthase and HIF-1*α* mRNA expression were measured with reference to the housekeeping gene *βA*. The TS : *β*-actin and HIF-1*α *: *βA* electrophoresis band intensity ratios were calculated for each tumour, and the mean ratio at each time point was expressed relative to time point zero.

### Statistics

Spearman's rank correlation coefficient (*r*_s_) was used to determine the correlation between marker expressions; the statistical significance was the two-tailed *P*-value for rejecting the hypothesis of zero correlation. Differences in marker expression during clamping were assessed using the Student's *t*-test.

## RESULTS

### Expression profiles of the hypoxic markers

**Figure 2:**
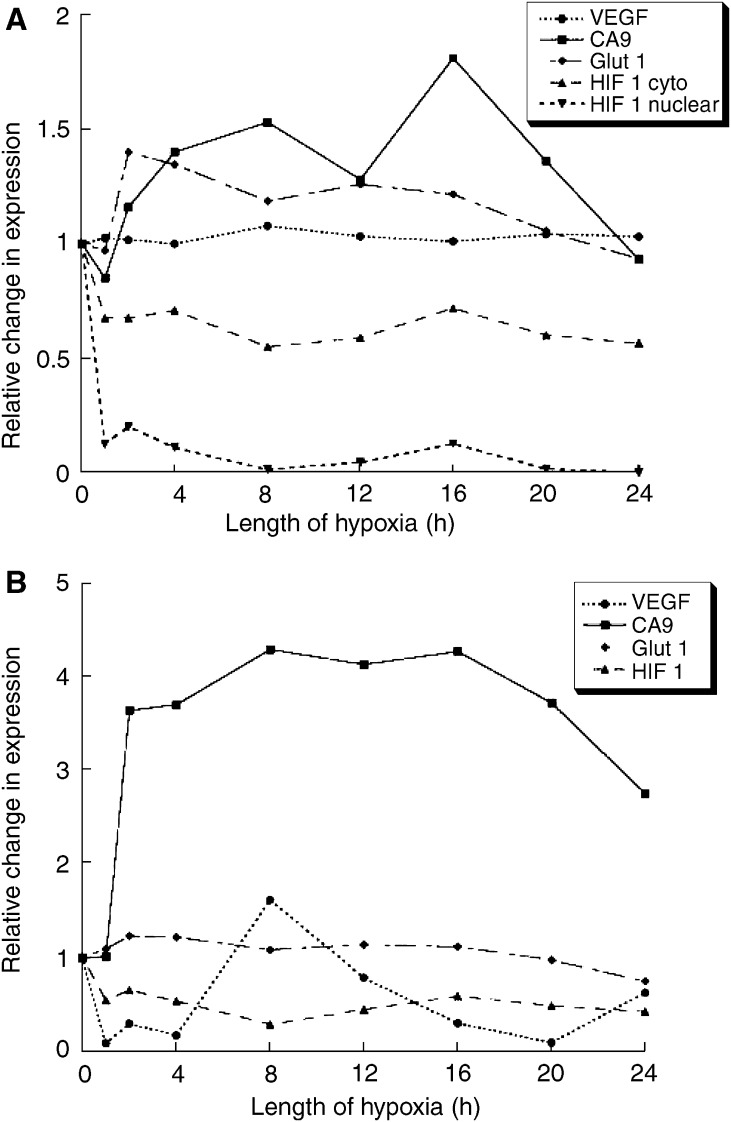
Stain intensity (**A**) and area (**B**) profiles for hypoxic markers.

For clarity, the expression of each protein during clamping was normalized to the time zero value as the OD varied considerably between the different markers. However, statistical significance was assessed on the mean OD values obtained from each of the 15 images from each time point. Figure 2A and B show that both HIF-1*α* stain intensity and area reduced during the first hour of ischaemia relative to the basal expression, and then remained at this level thereafter; all observations were significantly different to the preclamping value. The HIF-1*α* nuclear expression profile was similar to the cytoplasmic profile, but the reduction was more pronounced. Carbonic anhydrase-9 stain intensity gradually increased with prolonged ischaemia and became significantly greater than the preclamping level at 4 h (*P*=0.002). It reached a peak 1.8-fold increase at 16 h, before reducing to a level not significantly different to control levels at 24 h. The stain area increased four-fold after only 2 h of vascular occlusion (*P*<0.001), and remained significantly high thereafter. There was an early response in glucose transporter-1 (GLUT-1) stain intensity, with a maximum 1.4-fold increase after 2 h of ischaemia (*P*<0.001), which remained elevated until 20 h before decreasing towards control levels. There was a similar pattern for the area of tumour-expressing GLUT-1, which increased after 2 h (*P*<0.001) before returning to basal levels. Overall, clamping appeared to have little effect on VEGF stain intensity in this model, with a maximum 1.1-fold increase occurring at 8 h (*P*=0.03). Interestingly, VEGF stain area was significantly reduced throughout the experiment (*P*<0.001), apart from a spike occurring at 8 h.

### Expression profiles of the prognostic markers

**Figure 3:**
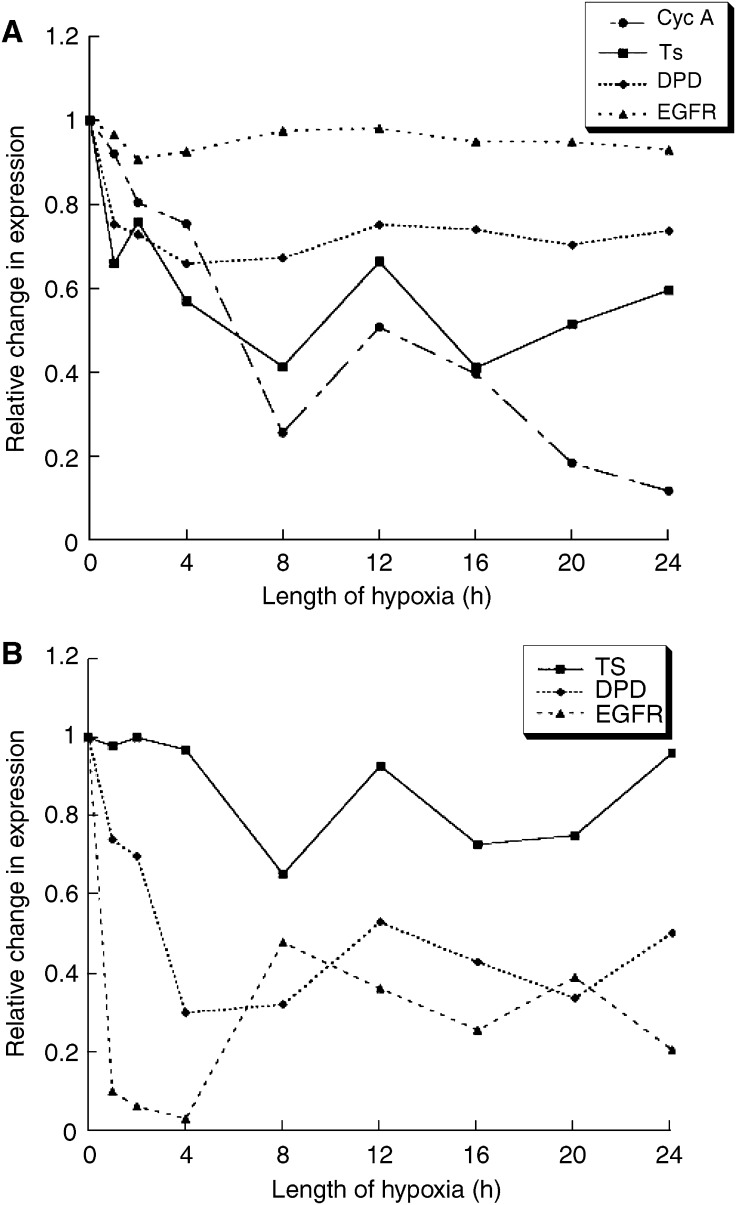
Stain intensity (**A**) and area (**B**) profiles for prognostic markers.

The prognostic marker data were analysed in a similar manner to the hypoxia-related proteins. Figures 3A and B show that the stain intensity for TS decreased rapidly after the first hour of ischaemia (*P*<0.001), and remained significantly lowered throughout the time course. The effect on area of tumour demonstrating TS expression was similar, becoming significantly low after 4 h (*P*<0.001). The expression profile for cyclin A followed that for TS, reducing throughout the experiment, and becoming significantly low after 4 h (*P*=0.03). Dihydropyrimidine dehydrogenase stain intensity and area both decreased following clamping (*P*<0.01) and remained low throughout. EGFR stain intensity was unchanged; however, the stain area reduced significantly during the first hour of hypoxia (*P*<0.001), and remained low thereafter, apart from a solitary spike at 8 h.

### Global changes in marker expression

**Figure 4:**
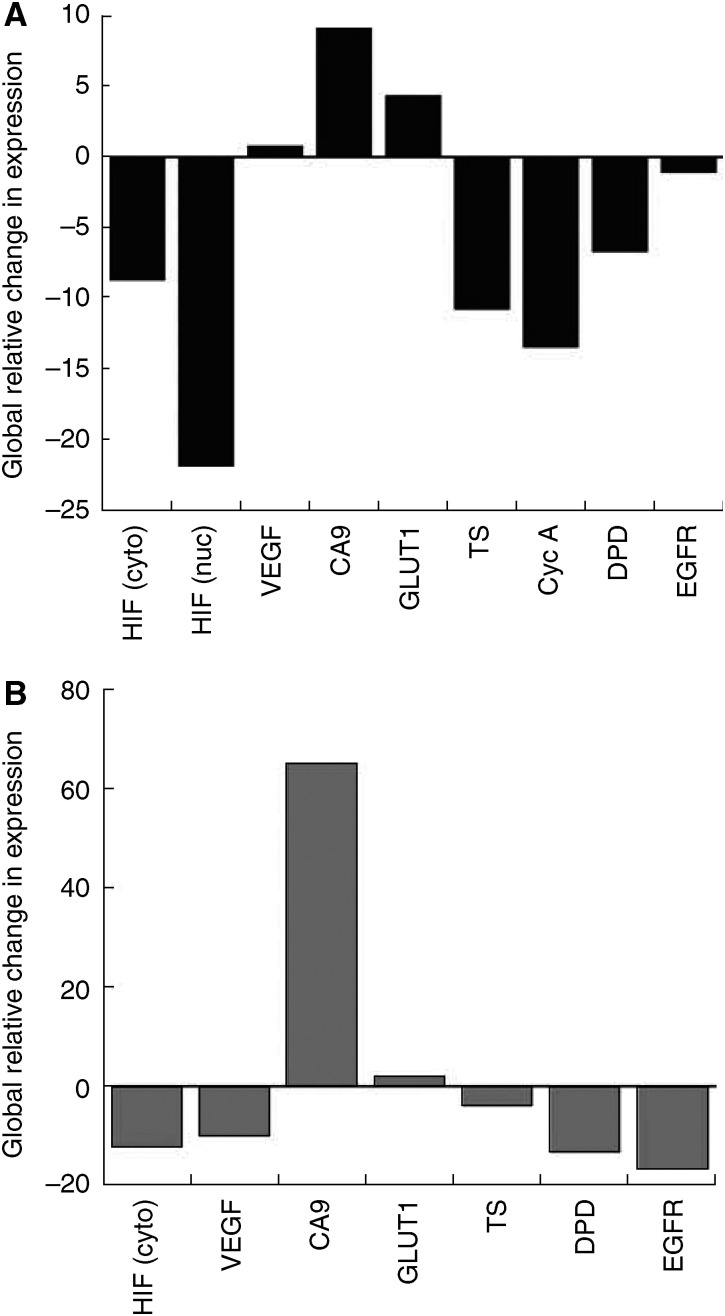
Global changes in marker stain intensity (**A**) and area (**B**).

Figure 4 illustrates the overall pattern of gene expression changes using area under the curve analysis. Carbonic anhydrase-9, GLUT-1, and VEGF all show variable increases in stain intensity secondary to vascular clamping, with CA-9 showing the greatest increase. The expression of the remaining markers all decreased, with HIF-1*α* nuclear staining showing the greatest reduction. For stain area, only CA-9 and GLUT-1 increased following vascular occlusion. The remaining markers all decreased, with EGFR having the greatest reduction.

### Changes in protein expression during the likely period of clinical ischaemia

The changes in expression occurring during the first 4 h are the most likely to be encountered in clinical practice. From Figures 2 and 3, it can be seen that CA-9 and GLUT-1 demonstrated increases of 40 and 35%, respectively, in stain intensity at 4 h, whereas TS and the nuclear HIF-1*α* count were reduced by 43 and 90%, respectively. For stain area, there was a 3.7-fold increase for CA-9, and decreases of 83 and 97% for VEGF and EGFR, respectively.

### Changes in mRNA expression for TS and HIF-1*α*

**Figure 5:**
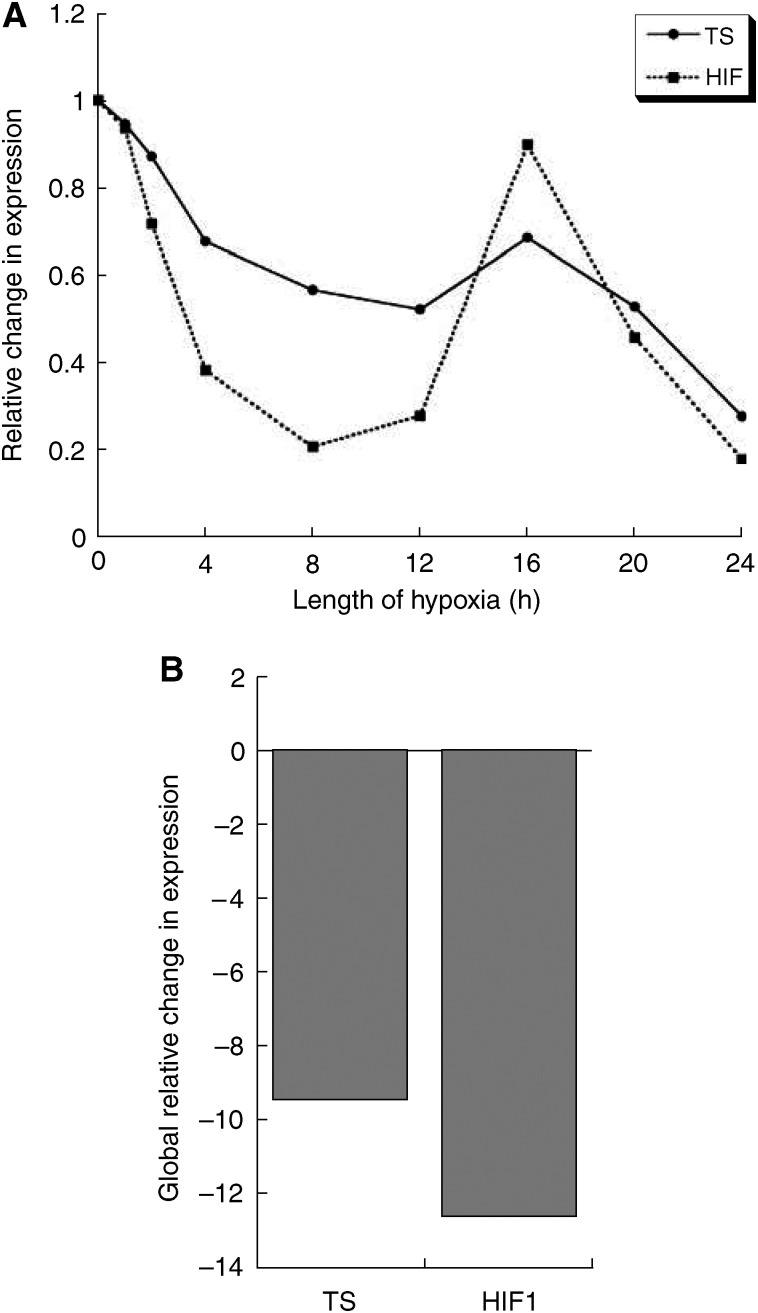
mRNA expression profiles for TS and HIF-1*α* (**A**) and the global changes in their expression (**B**).

It can be seen that the transcriptional profile for TS follows closely that of the translational response (Figure 5A and B), in that there is a sustained reduction in expression beginning after the first hour. HIF-1*α* mRNA expression was seen to reduce more rapidly than TS during the initial phase, before returning towards the basal levels after 16 h, and then demonstrating low levels of expression again at 24 h.

### Relationship between TS expression and markers of proliferation and hypoxia

There was a significant correlation between TS and cyclin A expression (*r*_s_=0.67, *P*<0.05), and an inverse relationship between TS and CA-9 expression (*r*_s_=−0.73, *P*=0.02), suggesting that changes in TS were related to changes in the proliferation rate, and that greater degrees of ischaemia were associated with greater downregulation of TS in this model.

## DISCUSSION

This study investigated the effect of surgically induced ischaemia on CRC gene expression. In this xenograft model, vascular clamping significantly altered the gene expression profiles of a number of markers suggested to be indicators of prognosis and response to chemotherapy. The model mimics *in vivo* tumour conditions closely, when tissues may be ischaemic for several hours following arterial clamping and before fixation. In fact, the temperature in these subcutaneous tumours approaches ambient within 1 h of clamping, with a protective effect on cell survival (Chaplin and Horsman, 1994). *In vivo* tumours will be at body temperature following clamping until they are removed from the body. Only then will the temperature approach ambient. There may be a much greater impact, therefore, on cellular processes, including gene expression, with *in vivo* warm ischaemia. Previous human studies have demonstrated temporal changes in mRNA expression for multiple genes within a microarray following extirpation of colonic mucosa, reportedly starting after 20 min (Huang *et al*, 2001). However, this was not confirmed by Sadahiro *et al* (2003) at the protein level, who found no difference in DPD activity at any time point following excision of colorectal tumour samples.

To investigate the degree of tumour hypoxia following vascular clamping, we measured the expression profiles of several endogenous hypoxia-related markers. Hypoxia-inducible factor-1 is a transcription factor involved in oxygen homeostasis (Zhong *et al*, 1999). The action of HIF-1 is to stimulate genes whose protein products act to increase oxygen availability or adapt to oxygen deprivation. These genes include erythropoietin, VEGF, GLUT-1, and CA-9 (Gillies *et al*, 2002). *In vitro* experiments have shown that HIF-1*α* increases rapidly within 30 min of a hypoxic stimulus, peaking at 2–4 h (Wiesener *et al*, 1998). However, we did not see an increase in HIF-1*α* expression in this model. This may be related to HIF-1*α* regulation by hypoxia-independent mechanisms (Kunz and Ibrahim, 2003) and/or greater HIF-1 degradation in totally anoxic tumours. In this xenograft model, tumours were exposed to chronic, or diffusion-limited, hypoxia, which HIF-1*α* reflects poorly (Janssen *et al*, 2002).

Hypoxia has been shown to induce CA-9 protein *in vitro* (Wykoff *et al*, 2000), and CA-9 has been shown to correlate with hypoxia measured by Eppendorf histography in carcinoma of the cervix (Loncaster *et al*, 2001). In our model, CA-9 expression showed the greatest increase for any marker, with a peak occurring after 16 h. CA-9 represents the response of tumours to chronic hypoxia (Hui *et al*, 2002), and our findings are in agreement with Lal *et al* (2001) who found CA-9 transcripts increased after 12 h of hypoxia in glioblastoma cells. Both GLUT-1 and VEGF have been shown to be upregulated in hypoxic conditions (Bashan *et al*, 1992; Paulding and Czyzyk-Krzeska, 2000). We found there was an early increase in GLUT-1 protein expression in response to surgically induced ischaemia; however, we did not see much variation in VEGF stain intensity. This is similar to Lal *et al* (2001) who found VEGF had one of the smallest *in vitro* hypoxic responses of a range of genes studied. The different expression profiles of the hypoxic markers studied in our model could be explained by their different susceptibilities to low oxygen and the rapidity of their response. In addition, the degree of necrosis in our experimental tumours following clamping is unknown, and the level of protein expression at any duration of ischaemia may represent the balance between hypoxic gene induction and protein degradation.

Previous experimental models of arterial occlusion have confirmed that changes in protein and mRNA expression do occur after variable periods of ischaemia. Rat testicular artery ligation for up to 6 h did not alter HIF-1 mRNA expression, but did lead to a two-fold increase in testicular HIF-1 protein expression after only 30 min (Powell *et al*, 2002); whereas rat middle cerebral artery occlusion led to an increase in HIF-1*α* and GLUT-1 mRNA in the ischaemic penumbra after 7.5 h (Bergeron *et al*, 1999). It may be that *in vivo* tissue ischaemia following vascular occlusion involves distinct responses to hypoxia and cellular energy depletion. A 45 min of rat renal ischaemia induced by vascular clamping was associated with increased HIF-1 and heat shock factor-1 (HSF-1) levels, whereas *in vitro* ATP depletion increased HSF-1 levels in isolation and hypoxia led to increased HIF-1 expression alone (Eickelberg *et al*, 2002).

The most widely studied prognostic and predictive marker in CRC is TS. We found surgically induced ischaemia had a pronounced effect on TS stain intensity, with a marked reduction in expression throughout the duration of ischaemia. Thymidylate synthase expression correlated with cylin A expression, suggesting that changes in TS were related to changes in the proliferation rate. Hypoxia slows the metabolic rate and is associated with lower levels of transcription and translation (Dachs and Tozer, 2000). Also, cell cycle arrest has been shown to be due to the hypoxic inactivation of nucleotide synthesis enzymes (Lando *et al*, 2002). It may be, therefore, the changes in TS expression seen in this model represent the changes in the cell proliferation rate secondary to hypoxia. Indeed, TS has been suggested to be a marker of proliferation (Derenzini *et al*, 2002), and *in vivo* studies have found a significant correlation with the expression of the proliferation marker Ki-67 in rectal cancer (Edler *et al*, 2002). We noted TS expression was related to the duration of ischaemia, and correlated inversely with CA-9 expression. It may be that the greater TS downregulation seen at these lower oxygen levels relates to the reduced cell turnover at this degree of hypoxia. Few studies have investigated the hypoxic response of the TS gene. An *in vitro* breast cancer study did report a reduced expression secondary to low oxygen tensions (Ehrnrooth *et al*, 1999); however, there was no correlation between TS expression and tumour hypoxia measured by Eppendorf histography in rectal cancer patients (Mattern *et al*, 1996).

The changes in TS and HIF-1*α* transcription were similar to their translational response, with a reduced mRNA expression following vascular clamping. Unlike the protein response, hypoxia has been shown to have little effect on HIF-1*α* transcription in cell culture (Giaccia *et al*, 2003). Similarly, TS is mainly controlled at the translational level (Liu *et al*, 2002), which provides an energy-efficient, rapid response to cellular stresses. However, we noted a sustained effect of ischaemia on TS and HIF-1*α* mRNA levels, highlighting the need for rapid preservation following tissue extirpation.

Dihydropyrimidine dehydrogenase is another putative prognostic marker in CRC. It is involved in the breakdown of uracil and thymine and is also the first, rate-limiting step in 5-FU catabolism (Beck *et al*, 1994). We found vascular clamping had a significant effect on both DPD stain intensity and area. Previous studies have found no correlation between DPD activity and time following extirpation of CRC samples (Sadahiro *et al*, 2003). Epidermal growth factor receptor is a member of the tyrosine kinase family of receptors and mediates the actions of several growth factors. It is frequently overexpressed in malignant tissues, and has been shown to be induced by hypoxia in a number of tumour types (Nishi *et al*, 2002; Gunaratnam *et al*, 2003). However, we did not see any effect of clamping on EGFR stain intensity, but there was an early decrease in stained area. As growth factor receptors are involved in cell division (Wahl and Carpenter, 1987), it may be the reduced expression seen that reflects the inhibition of cell proliferation occurring following tumour clamping.

The duration of tumour ischaemia most likely encountered in clinical practice would be up to 4 h. During this period, the arterial supply would be interrupted, the tumour would be resected, and the specimen fixed in formalin. Our data suggest that changes in gene expression do occur during this time frame. In particular, the expression of TS, CA-9, GLUT-1, and HIF-1*α* were all seen to alter considerably after 4 h, and these changes could be clinically relevant.

In this xenograft model, surgically induced tumour ischaemia altered the gene expression profiles of several markers purported to be indicators of prognosis and response to chemotherapy regimens in CRC, as well as estimates of tissue hypoxia. If these findings are reproduced in human tumours, they would have important implications on surgical technique and subsequent marker measurement. The results suggest that the length and degree of tumour ischaemia should be minimised prior to tissue fixation. This may be achieved by delayed vascular clamping or immediate fixation following extirpation. The findings also suggest the need for formal guidelines on rapidity of tissue fixation following surgical excision. Although CRC was the studied model, the results may be applicable to any tumour undergoing extirpation in which molecular markers have been proposed to dictate therapeutic strategy.

## Figures and Tables

**Table 1:** Markers studied, and conditions for immunohistochemistry

**Antibody**	**Pretreatment**	**Dilution**	**Source of antibody**
Thymidylate synthase (TS)	4 × 4 min microwave in citric acid with 10 min standing	1/300	Simon Joel, London, UK
Cyclin A	3 × 4 min microwave in citric acid with 10 min standing	1/100	#NCL-CYCLIN A, Novocastra Labs Ltd, UK
Dihydropyrimidine dehydrogenase (DPD)	4 × 4 min microwave in citric acid with 20 min standing	1/500	Masakazu Fukushima, Saitama, Japan
Epidermal growth factor receptor (EGFR)	4 × 4 min microwave in citric acid with 20 min standing	1/20	#NCL-L-EGFR, Novocastra Labs Ltd, UK
Carbonic anhydrase-9 (CA-9)	None	1/50	Adrian Harris, Oxford, UK
Glucose transporter-1 (GLUT-1)	3 × 4 min microwave in citric acid with 10 min standing	I/200	#A3536, DAKO Corporation, USA
Hypoxia inducible factor-1*α* (HIF-1*α*)	4 × 4 min microwave in citric acid with 20 min standing	1/1000	#ab463-100, Abcam Ltd, UK
Vascular endothelial growth factor (VEGF)	3 × 4 min microwave in Tris-HCL with 10 min standing	1/100	#MS-350-P1, NeoMarkers Inc., USA
